# Growing better brains? Pregnancy and neuroscience discourses in English social and welfare policies

**DOI:** 10.1080/13698575.2014.994479

**Published:** 2015-01-06

**Authors:** Pam Lowe, Ellie Lee, Jan Macvarish

**Affiliations:** ^a^ School of Languages and Social Science, Aston University, Birmingham, UK; ^b^ School of Sociology Social Policy and Social Research, University of Kent, Canterbury, UK; ^c^ Centre for Health Services Research, University of Kent, Canterbury, UK

**Keywords:** pregnancy, risk, family policy, neuroscience, maternal stress, foetal development, risk consciousness

## Abstract

In recent years, English welfare and health policy has started to include pregnancy within the foundation stage of child development. The foetus is also increasingly designated as ‘at risk’ from pregnant women. In this article, we draw on an analysis of a purposive sample of English social and welfare policies and closely related advocacy documents to trace the emergence of neuroscientific claims-making in relation to the family. In this article, we show that a specific deterministic understanding of the developing brain that only has a loose relationship with current scientific evidence is an important component in these changes. We examine the ways in which pregnancy is situated in these debates. In these debates, maternal stress is identified as a risk to the foetus; however, the selective concern with women living in disadvantage undermines biological claims. The policy claim of neurological ‘critical windows’ also seems to be influenced by social concerns. Hence, these emerging concerns over the foetus’ developing brain seem to be situated within the gendered history of policing women’s pregnant bodies rather than acting on new insights from scientific discoveries. By situating these developments within the broader framework of risk consciousness, we can link these changes to wider understandings of the ‘at risk’ child and intensified surveillance over family life.

## Introduction

In 2001, the Labour Government commissioned and developed a framework around the care and education for young children called ‘Birth to three matters’. The framework involved academic work as well as resources for early-years practitioners, and it was to compliment the Foundation Stage of the National Curriculum that set out educational aims for 3–5-year-olds. In 2010, the Cabinet Office published a report by Frank Fields, an MP and former Labour Government Minister, that recommended that the Foundation stage needed to be extended to include ‘the womb to five’ (2010, p. 6). In this article, we examine the ways in which the inclusion of pregnancy within the Foundation stage, with the accompanying understanding that the foetus is ‘at risk’ from pregnant women, has been influenced by specific understandings of the developing brain.

In this article, we explore emerging concerns over the foetus’ developing brain in UK government policy documents and discuss how this links to the construction of women as a primary risk factor. We argue that the current focus on pregnancy needs to be situated within the history of restrictions and advice given to pregnant women and normative constructions of ‘good mothering’. We show how the notion that pregnancy is a ‘critical period’ in brain development is used as a mechanism for getting access to families deemed to be ‘resistant to support’. Consequently, we argue that whilst the policymakers claiming that new scientific discoveries justify their approach, in reality such policies are a continuation of the gendered policing of women’s bodies.

## Risk, pregnancy and the development of the foetal brain

### Policing pregnancy

As Hays (1996) has noted, in the late twentieth century there was a change in expectations around motherhood. She argued that ideas about appropriate childcare intensified for middle-class women. Intensive motherhood involves seeing motherhood as a project in which women need to put their child’s needs first, and follow expert guidance on investing physically, emotionally and financially to ensure the best outcome. This is in contrast to earlier versions of ‘good motherhood’ in which keeping children fed, clean, warm and safe were emphasised more. Although individual mothering practices often vary from normative models, the idea of following a rational plan to try to maximise child development has nevertheless come to dominate understandings, whether or not it is embraced or rejected (Smyth, 2012). Moreover, the ability to ‘invest’ in the right form of parenting is dependent on a family’s socio-economic position (Gillies, 2005a; Nelson, 2010). Judgements about appropriate behaviour during pregnancy need to be understood within broader concerns of who is and who is not an appropriate mother (Rothman, 2014).

Pregnant women have long been subjected to surveillance and varying degrees of control over their behaviour, although how this is exercised changes over time (Oakley, 1986). The rise of ultrasound technologies has also reshaped the way that pregnancy is seen with increasing focus on the foetus’s welfare (Lupton, 2013). Moreover, understandings of the pregnant body need to be situated within broader changes associated with a rise of risk consciousness. Lee (2014) argues that risk consciousness in relation to parenting cultures has four interrelated elements. First, risk is now seen as an untoward possibility rather than a balance of probability. Second, risk is often individualised and generalised; children are often deemed to be ‘at risk’ rather than focusing on specific risky conditions. The third element is that ideas of risk are intertwined with or replace moral concerns. Finally, this new understanding of risk justifies the surveillance and policing of family life. Indeed as Lupton (2012) has argued, this is particularly true in relation to the pregnant body with the majority of concerns focusing on the foetus. As Lupton states:

Pregnant women are represented as the carriers of the precious foetus rather than individuals in their own right who have needs and priorities that may not always coincide with those of the foetus. (2012, p. 331)

This notion of the ‘at risk’ foetus, within a culture that emphasises individualism, means that pregnant women are expected to monitor themselves for potentially polluting substances and bodily states to enhance the developing foetus’s welfare (Lupton, 2012). In other words, the construction of women as foetal carriers divides the pregnant body and allows women to be positioned as potential abusers of the foetus.

The notion of the foetus as a vulnerable ‘citizen’ at risk from the pregnant body has emerged in conjunction with the rise of imaging technologies. As Lupton (2013) has shown, the foetus is now an iconic cultural image, often viewed as free-floating rather than located within a women’s body. Indeed the division between foetus and baby has becoming increasingly blurred as ideas of ‘rights’ and ‘citizenship’ come to be associated with the foetus (Lupton, 2012). It is in this context that attention and sanctions have increasingly focused on pregnant women. For example, in different parts of Europe, the guidance to pregnant women shifted to an abstinence position on alcohol consumption not because of any evidence that low-to-moderate consumption was harmful, but because it could not be proved completely safe (Leppo, Hecksher, & Tryggvesson, 2014; Lowe & Lee, 2010). In the US, legal action has been increasingly used against poor women in the name of foetal personhood leading to the imprisonment of women (Paltrow & Flavin, 2013). Both of these examples highlight the intertwining of moral judgements and assessments of risk that is an integral part of risk consciousness. It also connects with a new emphasis on the neurologically ‘damaged child’. For example, Teicher (2000) claims that physical, psychological and sexual abuse lead to permanent changes in a child’s brain, and suggests predictable negative outcomes. Yet there is extensive social science research that as Munro and Musholt (2013) have shown indicates that the impact of abuse varies and is influenced by complex social factors.

Ruhl (1999) has demonstrated that the responsibilisation of women produces notions of control and choice, yet pregnancy outcomes are not necessarily related to women’s will. The foetus cannot be reduced to the ‘product’ of pregnancy in a way envisaged by an increasingly actuarial society that emphasises accounting for costs and benefits. Ruhl (1999) argues that the shifting of responsibility to women’s behaviour ignores the complex interplay of biological and social factors, many of which are outside women’s control. Whilst pregnant women may understand and accept the general societal ‘duty to be well’, there is a tension with being able to comply with specific regimes in practice. Women can feel ambivalence about how others, such as health professionals and partners, seek to control or modify their behaviour (Ogle, Tyner, & Schofield-Tomschin, 2011).

Concerns about women’s behaviour during pregnancy are not new. The idea that women’s mental health during pregnancy has an impact on foetal development has a long history. For example, Shildrick (2000) has documented how both lay and medical texts in the eighteenth century raised concerns about maternal impression; the idea that a developing foetus could be physically or mentally ‘deformed’ through an ‘over-indulgence of fear or pleasure’ (2000, p. 255). Shildrick (2000) argues that central to these ideas are issues surrounding women’s moral and legal status; concerns about a lack of self-restraint merge into ideas about dangerous femininity. Consequently, whilst the suggested biological mechanism by which foetal development is affected may have changed, there is a need to consider how current concerns about maternal health are situated within a historical gendered framework on how the ‘other’ always poses a risk to ‘mainstream’ society. In other words, whether or not there are any proven biological mechanisms, it is important to understand the broader context in which these claims are made. It is into this complex situation that the ideas about the risk to foetuses’ brain development have emerged.

### Neuroscience discourse

As Wall (2010) has noted, during the 1990s ideas about brain capacity started to be noticeable in child-rearing discourse. Tallis (2011) makes an important distinction between neuroscience itself (the scientific study of brain function) and *neuroscientism*, which, he argues, is looking to understand humanity in the brain. Indeed as Rose and Abi-Rached (2013) have argued focusing on neural processes alone is reductionist and cannot explain, or predict, social life. In this section, we intend to briefly outline key ideas from neuroscience that are used in the policy. This is not intended to be a critique of neuroscience itself, but to enable the policy-claims around brain development to be contextualised.

As we have argued elsewhere (Lowe, Lee, & Macvarish, in press), a specific understanding of early intervention in child welfare policies seems to be built around a reductionist argument of brain development. A central idea is that during the early years of a child’s life, normal development will be disrupted unless the correct ‘environmental influences’ ensure proper neural development. An important element to this concept is the idea of intergenerational transmission, that people’s ability to be good parents relates to their early childhood. The implication is that today’s ‘bad’ parents act in this way as their brains were not developed properly, and thus they cannot be expected to raise their children adequately unless interventions can override ‘fixed’ biological constraints.

Bruer (1999) has shown how this understanding of brain development arises from a particular set of ideas that have been associated with early childhood. These are developmental synaptogenesis, sensitive periods and enriched environments. Bruer (1999) argues that whilst it is the case that developmental synaptogenesis (rapid increase in synapse density) takes place in the early years this is not the only time this happens. Moreover, more synapses do not necessarily mean additional brain functionality. In other words, it is not clear that this process is linked to the future potential of children. Bruer (1999) also notes that the idea of sensitive periods has been exaggerated. There are a small number of skills that are time sensitive in terms of development; these are what he calls ‘species-typical’. This means that it would be extremely unlikely not to encounter them within the general environment. For example, whilst animal experiments have shown that depriving young of vision or from hearing certain sounds does affect their development, it would be a rare occurrence that children were in a similar position. For example, Hubel and Wiesel (1970) demonstrated that if you sewed newborn kitten’s eyes shut, it had a long-term impact on their vision. However, as Bruer (1999) points out, that it is unlikely that this level of deprivation would ever occur naturally, and most eyes would be exposed to different forms of light needed for development even in quite adverse circumstances. Bruer (1999) notes that species-typical development issues are exceptions and the majority of skills and behaviours are learnt throughout life rather than dependant on exposure in the early years.

The third aspect of Bruer’s (1999) critique is around what qualifies as sufficient environment for development. Whilst very extreme neglect can lead to altered brain development, he argues that this is not the same as saying that enriched environments will develop better brains. Much of the work in this area has looked at the development of children who were subjected to extreme neglect in Romania orphanages. For example, Behen, Helder, Rothermel, Solomon, and Chugani (2008) found 46% of children who had experienced early severe deprivation had impairment in specific cognitive domains. Yet as Rutter et al.’s (2010) work has shown, there could be good recovery even after early extreme neglect and that *institutional* deprivation was the key determinant of development issues. Hence, whilst being raised in a ‘dysfunctional’ family may be problematic; this is unlikely to lead to some form of brain deficit (Bruer, 1999; Wastell & White, 2012).

Despite well-established critiques, the three interlocking ideas of developmental synaptogenesis, sensitive periods and enriched environments have been used to position the mother and/or parenting as a crucial biological determinant of future lives. Examples of brain-claims are highlighted in O’Connor, Rees, and Joffe (2012) study of brain and child development ideas in the media. They found issues such as sexual orientation and the risk of psychiatric disorders being linked to the intra-uterine environment. These brain-claims are part of a broader trend of claims about the ‘biological embedding’ of the mother’s health on the developing foetus within an area often referred to as ‘foetal programming’ (Axford & Barlow, 2014). Whilst clearly there is a link between the health of the pregnant women and the foetus, a simplistic deterministic mechanism is likely to overlook the complexity of both biological mechanisms and social lives. Moreover, the idea that brain development is at risk from the pregnant body justifies health and welfare interventions. The resulting logic is that interventions should be as early as possible, preferably before conception, because of this specific construction of the early years as having a unique and deterministic impact on later life chances through the developing brain. Hence, biological claims are a crucial element in the way that risk consciousness operates in this area.

Lawless, Coveney, and MacDougall (2014) found that there is growing interest in infant mental health. They argue that the language of brain development has been used to update ideas of attachment set out by Bowlby. Within the broad framework of attachment theory, the emotional bonds that an infant develops with a primary caregiver (usually their mother) are central to their future emotional health. As detailed assessment of infants themselves is not possible, it is mother’s behaviour that is often monitored for potential concerns (Lawless et al., 2014). They argue that discourses of individualised risk dominate professional understandings of infant mental health and minimise attention on structural issues such as poverty.

This particular understanding of brain development as deterministic of life chances has had a profound impact on recent policy development. Wastell and White (2012) have shown in the area of child welfare that there has been a shift towards medicalised interventions in family life to try to ‘cure’ the problems. Featherstone, Morris, and White (2013) have documented the way this has led to a shift from family support to a specific form of child protection, with the notion that quick removal of children is always preferable. Both these articles provide evidence for the ways in which brain development arguments are becoming central to the identification of children ‘at risk’ and the potential deficits that need targeting in family life. Moreover, as we have argued elsewhere (Lowe et al., in press), this particular form of understanding leaves little capacity for children to be considered as autonomous individuals who are active agents in their own lives. We draw on data in this article to investigate how neuroscientific discourse are reshaping ideas about pregnancy within the English welfare policies and the extent to which the operation of risk consciousness within neuroscience discourses are producing particular understandings of foetal risk.

## Methods

In this article, we draw on data from a study of the emergence of ‘brain-based’ understandings of child development in English social and welfare policies around parenting. The aim of the project was to trace how concepts and language taken from neuroscience were used within policy to explain parent–child relationships and justify specific interventions in family life. In it we analysed a purposive sample of 42 documents from 1998 to 2012 to identify how ‘neuroscientific evidence’ was used in policy thinking.

We identified potential policy documents through searches of government websites and other records of policy development. The majority of policy documents are a matter of public record and freely available to access. Our final selection of documents for analysis was based on three criteria. We included documents if they: contained an in-depth discussion of key concepts (such as parenting, brains); covered a range of social and welfare areas (e.g., social exclusion, health and maternity services); or appeared to be significant in the development of later policies.

We sought to map a chronological account of how neuroscience discourses emerged as a significant issue in policy understandings of health, child development, parenting and early-years childcare. Our final sample of documents included: policy consultation and strategy documents published by Government departments (29); reports commissioned by government departments (7); and reports by think tanks and advocacy groups that were key reference points in later policy (6). Full details of the sample can be found in Lee, Macvarish, and Lowe (2013). We thematically analysed the selected documents, a process that involved, close reading, coding and the mapping of key terms and ideas. Members of the research team read the documents and developed an initial coding framework. This was refined during the analysis period through ongoing discussion within the research team until we were confident that it reflected the issues in the data set. The coding was then used to develop an understanding of the emerging themes. This analysis was facilitated by NVIVO. This method of policy analysis is similar to Leppo et al. (2014).

In this article, we focus on one aspect of this analysis, how these brain discourses construct specific understandings of pregnancy. We use quotations from the documents that best illustrate the trends in the policies.

## Findings

### The ‘problem’ of pregnancy

We start our analysis of the documents by focussing on three documents that illustrate the development of the neuroscience discourse. Following their election in 1997, the New Labour government in the UK introduced a range of policy initiatives under a broad aim of trying to ‘support’ parents (Gillies, 2005b). The range and scope of their policies, which were often contradictory in their aims, mainly addressed issues relating to concerns about crime and disorder (Gillies, 2005b). In this period, there is very little emphasis on the ‘quality’ of women’s pregnancies. For example, in *Supporting Families* (Home Office, 1998) pregnancy is mentioned or alluded to in three main ways: teenage pregnancy, pre-natal education and maternity leave. The most prominent issue is the concern about the ‘problem’ of teenage pregnancies. In the Home Office policy document teenage pregnancy was identified as a ‘serious’ problem but the problem was framed as a social exclusion issue not as one of physical risk to the developing foetus as can be seen here:

Unwanted and under-age pregnancies, whether planned or unplanned, have a high personal, social and economic cost and can blight the life chances of younger teenagers. Many already vulnerable groups are disproportionally likely to become teenage parents, increasing the chances that they and their children will continue to be affected by social exclusion. (Home Office, 1998, p. 44)

The other two ways pregnancy enters the document similarly lack an emphasis on the developing foetus. The main aim of the document was to highlight and extend the role of parenting support, and within this document, parenting begins at birth. This can be seen through the lack of attention to the pre-natal period as an essential time for intervention, instead stating that women may require support ‘around the birth of their children’ (Home Office, 1998, p. 11). In relation to maternity leave, there is discussion about extending the legal time periods, but no concerns about pregnancy are made specifically. This example illustrates that at this time, the developing foetus was not a significant focus of family policy, and in general, the need for ‘good parenting’ appears to begin after birth.

By 2003, the emphasis has changed as can be seen in a Department for Education and Skills Research report ‘Birth to three matters’ (David, Goouch, Powell, & Abbott, 2003). This report was a review of literature commissioned as part of the introduction of the *Birth to Three Framework*. The framework was designed to provide guidance for those who were working as childcare practitioners, and the supporting documentations such as ‘Birth to three matters’ was intended to provide the supporting evidence for such practice. Most of ‘Birth to three matters’ is about early childhood, but it also discusses some pregnancy issues that may impact on the health of the foetus such as nutrition, smoking and use of illegal substances (David et al., 2003). In the sections on pregnancy, ‘Birth to three matters’ identified two key areas that were to dominate ideas about pregnancy in subsequent policy documents: maternal stress and brain development.

‘Birth to three matters’ discussed two studies which indicated that pregnant women’s mental health could have an impact on the developing foetus. The document outlines Grossman et al.’s study which showed that stress during pregnancy can lead to a more active foetus and newborn irritability (Grossman et al. in David et al., 2003, p. 58). However, ‘Birth to three matters’ notes that such evidence does not necessarily predict there will be any long-term issues in childhood. Furthermore, in ‘Birth to three matters’, the discussion of the Grossman et al.’s study is in a section on ‘special babies’ and there is no mention in this section of the developing brain as an issue. However ‘Birth to three matters’ also cites a study by Eliot that suggests that not only does maternal smoking and drinking have ‘detrimental influences’ on brain development but maternal emotion and stress does as well (Eliot in David et al., 2003, p. 122). Overall ‘Birth to three matters’ is tentative about claims that foetal brain development can be harmed by maternal behaviour and states that neuroscience researchers ‘are cautious about claims that certain experiences or products can boost brainpower or make long term differences’ (David et al., 2003, p. 14).

However, by 2011 there was a marked change as the discourse of neuroscience became more generally embedded in policy documents (see Lee et al., 2013), and pregnancy was increasingly defined as a key site of potential intervention in developing brains. This can be illustrated in Allen’s (2011) report published by the Cabinet Office, ‘Early intervention: the next steps’ that included the following statement:

Although poor parenting practices can cause damage to children of all ages, the worst and deepest damage is done to children when their brains are being formed during their earliest months and years. The most serious damage takes place *before birth* and during the first 18 months of life when formation of the brain governing emotional development has been identified to be taking place. (…) Psychosocial stress during pregnancy has been linked to increased risk for attention deficit hyperactivity disorder, schizophrenia and social abnormalities. (Allen, 2011, p. 15, our emphasis).

This quotation comes from a chapter entitled ‘Using our brains’ that claimed to set out the rationale behind the government’s early intervention policy. The central rationale was that that adult ‘dysfunctions’ (such as crime, alcohol and/or substance misuse) are related to the inferior architecture of the brain that are formed during the early years. Importantly, this extract from the policy document shows how the mother’s emotional state was deemed to be a crucial determinate. Pregnancy was clearly identified as a time when maternal stress could negatively affect a child’s future life. Thus, ideally interventions to reduce social problems needed to begin *before* conception.

These documents illustrate how in a relatively short space of time, there has been a shift in policy concerns from the age of some mothers (teenage pregnancy) to the *quality* of the pregnancy itself. Whilst issues such as substance use have been present for some time, a new emphasis on women’s mental health during pregnancy has been constructed as a significant risk to the developing foetus. This risk includes women already deemed to be poor mothers, such as young women (Allen, 2011), but also potentially generalises the risk to any woman subjected to stress during pregnancy. This duality of highlighting specific individuals, often within a moral framework, alongside a potential generalising trend illustrates the features of risk consciousness that are operating in the area.

### Maternal mental health

In some of the earlier policy documents, whilst maternal stress was highlighted as a risk factor for the developing foetus, it was acknowledged that the casual mechanism was unclear. For example, in a Department for Education and Skills briefing document, *Support from the Start* (Sutton, Utting, & Farrington, 2004) there was a section on stress during pregnancy and it cited a number of studies that claimed that there was evidence that behavioural problems, in general, and attention deficit hyperactive disorder, in particular, could be linked to stress during gestation. *Support from the Start* acknowledged that the biological process was not known and noted that there were currently no studies that have demonstrated how to reduce stress during pregnancy, although it suggested that family support interventions might be helpful. Whilst not specifically mentioned in this section of the document, it becomes clearer later that the interventions were only for specific women; those in disadvantaged neighbourhoods or of a younger age. Hence, the emphasis was not on the general impact of maternal stress but on the impact on specific categories of pregnant women for whom intervention might reduce risk to the developing foetus.

By 2008, there was no longer any uncertainly that maternal anxiety and depression had an adverse impact on neurological development of all foetuses. The Department of Health policy document on *Pregnancy and the ﬁrst ﬁve years of life* in its child health promotion programme summarised the evidence that maternal anxiety and depression had an adverse impact on the foetal and baby’s brain in the following way:

The CHPP [Child Health Promotion Programme] needs to reflect new evidence that has emerged about neurological development (…). It should also incorporate the information that we have about the adverse effect that maternal anxiety and depression in pregnancy can have on child development. A child’s brain develops rapidly in the first two years of life (…). Early interventions directly affect the way that the brain is wired, and early relationships set the ‘thermostat’ for later control of the stress response. This all underlines the significance of pregnancy and the first years of life (Department of Health, 2008, p. 9)

This approach is typical of policy documents of the period in that it fails to give concrete evidence of how the neurological development of the foetus is affected. Like other policy documents of the period, *Pregnancy and the ﬁrst ﬁve years of life* made reference to ‘new understandings’ about the relationship between maternal stress and brain development but it was not clear what these were and what evidence they were based on so it is difficult to properly assess such claims. Furthermore, these policy documents tended to merge pregnancy and the post-natal period together as if there was no significant difference between the two.

Another document from this period *Early Intervention: Good Parents, Great Kids, Better Citizens* (Allen & Duncan Smith, 2008) was not an official government policy document but is important as both authors were to become involved in policy formation after the election of the Coalition government in 2010, Allen as author of a Cabinet Office policy document and Duncan Smith as Secretary of State for Work and Pension. Allen and Duncan Smith outlined a clear relationship between stress and the development of the brain in the following way:

If a child’s early experience is predominantly characterised by fear and stress, then the neurochemical responses to fear and stress *become the primary architects of the brain*, for the simple reason that these are the responses most frequently triggered. The stress hormones, such as cortisol, that are elevated during trauma, flood the brain like acid (Allen & Duncan Smith, 2008, pp. 58–9 original emphasis)

Allen and Duncan Smith claimed that this adverse brain reaction led to a range of unhealthy and antisocial behaviours such as educational failure, crime and illegal drug use (Allen & Duncan Smith, 2008). In other words, stress needed to be avoided for the benefit of wider society as much as the individual.

As briefly mentioned earlier, within the policy documents of this period there is often a disregard of the difference between pregnancy and the post-natal period. This means that the inference is that stress during pregnancy will therefore have a similar impact on the foetus as it does on a young developing child. For example, a Cabinet Office (2006) document on social exclusion, *Reaching Out: An Action Plan on Social Exclusion*, combined the two periods in the following way:

Poor parenting can expose children to greater risks and can contribute to the development of potentially harmful patterns of behaviour. Intensive support can improve parenting and attachment, and can have dramatic impacts upon parent and child outcomes. (…). Pregnancy and birth is a critical time, when it is possible to develop the resilience and protective factors in children, thereby dramatically improving their chances of going on to lead healthy and fulfilling lives (Cabinet Office, 2006, p. 46).

The overwhelming messages in the more recent policy documents is that mothers need to reduce stress and bond with their babies before birth in order to prevent adverse brain development. The policymakers in an era of austerity are concerned about short-term and long-term cost reduction, for example preventing the development of antisocial adults as well as enhancing child welfare. There is in these documents little discussion of the welfare and health of pregnant women and the mothers of newborn babies.

Another important element in the policy documents is stress in pregnancy emerges as a social class issue. While the documents cite the mediating role of biological mechanisms of hormones such as cortisol, these mechanisms are treated differently in terms of different social classes. The policy documents focus on stress and damage amongst disadvantaged women, and identify these as social problems that require policy action. This emphasis is rarely explicit in the documents and is most clearly articulated in relationship to the ‘intergenerational’ transmission of antisocial behaviour. For example, in Department for Education’s (2011, p. 18) policy document on families with young children brain development was identified as a key part of child development and future outcomes. The document identified an intergenerational cycle that needed to be broken by early intervention:

While all families benefit from help at some point, for some the need is more acute. (…). It is now very clear that early help and intervention is crucial if we want to support families to get out of a cycle of poor outcomes that repeats itself over and over through the generations. (Department for Education, 2011, p. 2).

The central message contained in these later policy documents is that stress or depression in disadvantaged women is a causal factor in adverse foetal brain development leading to antisocial behaviour in adults. Yet there is not a similar concern over the mental state of more affluent women. In other words, stress in middle-class women’s lives does not seem to have the same biological impact. This has resonances with the ideas of the dangerous ‘other’ (Gillies, 2005a).

### Critical periods

The policy documents argue that potential harm to foetal and infant brains can be minimised if interventions take place before fixed or sensitive periods. In relation to brain development, the notion is that unless the ‘correct’ stimulation takes place, then the brain will lose its main chance for a particular development process. For example, in Allen’s (2011) ‘independent report’ for the government on early intervention published by the Cabinet Office identifies ‘sensitive windows of time’ in the following way:

Different parts of the brain (governing, for example, sight, hearing, etc.) develop in different sensitive windows of time (…). Studies show maternal depression is a prime factor in determining behavioural problems for many children and impedes brain development (…). These emotional deﬁcits are harder to overcome once the sensitive window has passed. (p. 16)

In this statement there is a sleight of hand that extrapolates critical periods of development for specific brain functions such as vision into general development of all functions partly through the use of the word, ‘etc.’ (see Bruer, 1999 for a detailed assessment on how different aspects of vision sensitivity occur up to the ages 8 or 9 if not beyond). The inference in this statement is that all areas of future life are influenced by pregnant women’s mental health. Furthermore, the document fails to note the evidence that time-sensitive issues are related to a minority rather than the majority of child development issues (Bruer, 1999). The document also does not actually give full details about the type of conditions, such as the orphanages in Romania, that have been shown to lead to significant impairments (Behen et al. (2008) and instead uses terms such as ‘infant trauma’, ‘abuse’ and ‘neglect’. As maternal stress is constructed as ‘risk factor’ for these, the foetus ‘at risk’ is democratised from extreme circumstances to any pregnancy where the woman is stressed.

In many of the policy documents, the notion of critical periods was used to define pregnancy as a key time that women were receptive to interventions. In the Department of Health (2011, p. 4/12/109) document *Preparation for Birth and Beyond,* this critical period was defined as ‘window of opportunity’:

There is now greater understanding of how psychological factors can impact on a baby’s development in the womb. Pregnancy is a window of opportunity, as parents are receptive to learning and making changes.

The discussion of such critical period in documents is framed in terms of women’s social circumstances and the impact if these on their mental health. For example, Allen and Duncan Smith in their think-tank review in 2008 argued that during this period it is possible to access hard to reach groups making interventions during this period particularly important:

This is a critical intervention, not least since many of the hard-to-reach individuals who are, at any other time, most resistant to public authority will respond when pregnant to a friendly and helpful midwife or health visitor who can then open the door to others later who may help, for example, with training or education. To put it in economic terms, it is the best investment opportunity in our human capital: all later investments are more expensive, riskier and give diminishing returns. (*Early Intervention: Good Parents, Great Kids, Better Citizens* 2008, p. 77).

Whilst the designation of ‘hard to reach’ can cover different social groups, given the framing of the rest of the document, this was implicitly women from lower social economic classes. The justification presented is about the cost-effectiveness of protecting future children. However, as the ‘risks’ only appear to be of concern about ‘othered’ women, we would argue that this needs to be understood as a moral judgement rather than just a medical concern.

## Discussion

The ways in which recent policy documents use the concept of critical period as a time frame for both brain development and pregnancy and as a way of identifying a point of intervention indicate that neuroscientific discourses have become central to the ways in which family policy is written. Crucial to both brain development and pregnancy is an understanding of population vulnerability. Foetuses are ‘at risk’ because their brains are developing rapidly, and pregnant women, especially those from lower social classes, need to be supervised by the state to ensure that they do not damage the foetus during these critical periods. Moreover, this policy has developed at a point in time where we have a ‘window’ on the developing foetus through scanning technologies, and this has shifted our understanding of the foetus towards personhood if not citizenship, discursively separating them from their mother’s bodies before they are born (Lupton, 2013).

In a recent paper on policy and practice around neurodevelopment in children’s early years, Glaser (2014) states that brain development is harmed pre-natally by significant maternal stress. She references Welberg and Seckl (2001) but fails to point out that this was an animal study and they acknowledge that as brain development is different in rats, their study is not directly transferrable to humans. This slippage from what science has actually found and the way that it is used in policy development is hugely problematic. It is also the case that many of the policy documents making claims about new scientific knowledge omit the evidence, making it difficult to assess the validity of the claims. Taken together, this misuse and missing evidence suggest that these claims are a continuation of the gendered history of policing pregnant women (Hallgrimsdottir & Benner, 2014) rather than newly discovered risks to the developing foetus. Moreover, this needs to be situated within the broader framework of risk consciousness that surrounds family life (Lee, 2014)

Broader changes in the construction of ‘good motherhood’ in which intensive motherhood has come to dominate understandings (Hays, 1996) alongside the shift to an actuarial society within which women are held responsible for the outcome of pregnancy have contributed to motherhood as beginning prior to birth and the distinction between foetus and baby becoming blurred (Lupton, 2013). Indeed as Munro and Musholt (2013) have noted some policymakers are using neuroscience as a rhetorical device to add weight to political arguments. In relation to pregnancy, we would argue that whilst the idea of (adverse) brain development is stated as the rationale, it is the underlying political concerns about potential societal disorder from poor women’s children that is at the heart of these policies. Risk consciousness allows these moral concerns to be individualised onto the ‘vulnerable foetus’ rather than seem as part of the history of gendered policing of (some) women’s bodies. The focus on biological rather than social understandings also needs to be placed within a broader framework in which children’s development is seen as largely determined by adults (Christensen, 2000).

The idea of ‘critical periods or windows’ for intervention illustrates the extent to which neuroscience discourse has entered the policy arena. As we have argued elsewhere, the notion that all future potential of a child is limited by what happens to their brain in a few specific moments in early life is clearly infant determinist (Lowe et al., in press). The ultimate implication is that should ‘non-optimum’ conditions be present during the early years then there is little hope of recovery. This idea is not only depressing but also has profound implications. For example, the Children and Families Act 2014 has a reduced emphasis on family care in favour of increasing forced adoptions and allows children to be placed with potential adopters before any court proceedings have taken place (Ashley, 2014). Whilst ideas about neuroscience are not the only reason for this change, they are part of the current context of child protection (Featherstone et al., 2013). These developments are a clear indication of the shift in understandings of risk as untoward possibilities rather than balance of probabilities.

The increased emphasis on pregnancy as an essential part of the ‘critical window’ means that the gaze of policymakers is clearly focused on the intimate habits and dispositions of the mother. This extends beyond the physical surveillance of women’s bodies and behaviour into their minds as their mental state becomes named as a key determinate of foetal health. It is no longer enough to eat by the rules and abstain from activities deemed to be hazardous, (poor) women must not become stressed. However, these policies have been developed alongside serious cuts to the welfare state, within which welfare-dependent troubled families are deemed to need tougher rather than more generous welfare support (Slater, 2014). Arguably, increasing levels of poverty are likely to increase rather than reduce the stress levels in poorer pregnant women.

In this article based on a study of policy documents, we have identified similar trends to the ones which Lawless et al. (2014) found in their study of the Australian professional understandings of infant mental health. They argue that:

Threads from discourses of brain science, attachment theory, critical periods, children’s needs, mothering and maternalism have woven together to shape thinking and practices around infant mental health in often invisible ways. (Lawless et al., 2014)

The notion that pregnancy is not just a ‘critical window’ in terms of foetal development, but also a key temporal point for intervention is also of key importance. These policies use neuroscience to justify the idea that early intervention is a cost-effective way of addressing social problems and subjecting women, especially ‘risky’ women, to surveillance and control during pregnancy is a way of furthering these policy objectives. By framing the ‘problems’ as inherently biological attention is drawn away from the continuities of social condemnation of particular pregnancies. Yet in a time of increasing hostility towards poorer families in addition to starker child protection policies, it is not clear that many pregnant women will be in a position to refuse increasing surveillance and intervention. The attention on the pregnant body needs to be understood within a wider change in which risk consciousness has come to dominate policy development over family life. As Leppo et al. (2014, p. 525) argued alongside the cultural image of the foetus, are the twin images of the ‘perfect mother’ who follows expert advice and the ‘failed’ mother who has ‘taken chances’ with her child’s future health. These pressures illustrate the normalised scrutiny of pregnancy and increasing discourse of foetus as citizen.

## Conclusion

In this article, we have explored the emergence and significance of ideas about pregnancy and the developing brain within British family policy within the broader framework of risk consciousness. This renewed emphasis on biology is used to potentially increase the policing and surveillance of family life. We have shown how there has been a shift from concerns about the age of motherhood to the quality of the pregnancy itself and that this is symptomatic with a growing emphasis on the ‘vulnerable foetus’. In recent documents, maternal stress is identified as a risk to the developing foetus and this has the potential to generalise to a wider population. Yet the emphasis on women living in disadvantage undermines the claims to biology through its selective application and re-individualises the risk. Hence, moral concerns about particular pregnancies are reconstructed as risk, particularly through the rhetoric of ‘critical windows’. Yet the social element, women’s openness to compliance with interventions, rather than the neurological element seems to be the critical point. Consequently, we would argue that these policies need to be understood as part of broader trends of policing of women’s bodies in the name of the foetus rather than acting on insights from new scientific discoveries.
